# SDS-Assisted Morphology Regulation of NiMoO_4_ for Efficient Alkaline Water Oxidation

**DOI:** 10.3390/molecules31183227

**Published:** 2026-09-12

**Authors:** Mrunal Bhosale, Pritam J. Morankar, Sahil S. Magdum, Aditya A. Patil, Chan-Wook Jeon

**Affiliations:** School of Chemical Engineering, Yeungnam University, Gyeongsan 38541, Gyeongsanbuk-do, Republic of Korea

**Keywords:** oxygen evolution reaction, NiMoO_4_-SDS, transition metal oxides, overpotential, electrocatalyst

## Abstract

Oxygen evolution reaction (OER) remains the primary kinetic bottleneck in alkaline water electrolysis, necessitating the development of efficient, economically viable, and earth-abundant electrocatalysts. Herein, a facile sodium dodecyl sulfate (SDS)-assisted hydrothermal strategy is employed to synthesize nanostructured NiMoO_4_ electrocatalysts with tailored morphology and enhanced electrocatalytic activity. Acting as a soft-template and structure-directing agent, SDS regulates the nucleation and growth of NiMoO_4_, resulting in a porous and interconnected nanostructure with abundant exposed active sites. The optimized NiMoO_4_-SDS-2 electrode exhibits outstanding OER performance, requiring an overpotential of only 428.5 mV to achieve a current density of 100 mA cm^−2^, together with a Tafel slope of 62.4 mV dec^−1^, demonstrating accelerated reaction kinetics. Electrochemical impedance spectroscopy and double-layer capacitance analyses reveal enhanced charge-transfer capability and a larger electrochemically active surface area, while long-term durability tests confirm excellent operational stability under alkaline conditions. The enhanced catalytic performance is associated with SDS-induced morphology regulation, improved active-site accessibility, and favorable interfacial charge-transfer characteristics. This work demonstrates that surfactant-assisted soft-templating is an effective strategy for engineering high-performance NiMoO_4_-based electrocatalysts and provides valuable insights into the rational design of advanced transition metal oxide catalysts for efficient and durable alkaline water oxidation.

## 1. Introduction

The urgent global shift toward sustainable energy is driven by accelerating environmental degradation and the depletion of finite fossil fuel reserves. Within this framework, electrochemical water splitting has emerged as a cornerstone technology for carbon-neutral energy conversion [1,2]. By decoupling water into its constituent elements via hydrogen evolution reaction (HER) and oxygen evolution reaction (OER), this process yields high-purity hydrogen with zero localized emissions [3,4]. Because it can be efficiently integrated with intermittent renewable sources like solar and wind, water electrolysis represents a technically robust pathway for establishing a viable, large-scale hydrogen economy. While noble metals such as Pt, IrO_2_, and RuO_2_ remain the gold standard for HER and OER owing to their exceptional kinetics, their scarcity and high cost severely limit large-scale industrial use [5,6,7]. Consequently, there is a pressing demand to engineer earth-abundant, cost-efficient alternatives that provide comparable catalytic activity and long-term durability to drive the next generation of energy-conversion technologies.

Transition metal oxides (TMOs) have emerged as promising candidates for electrocatalytic water splitting, especially for the OER. This growing interest is driven by their earth-abundance, cost-effectiveness, and impressive balance of catalytic efficiency and long-term structural stability [8,9]. The electronic versatility of transition-metal oxides, arising from their multiple oxidation states and tunable metal-oxygen coordination, plays an important role in regulating OER activity. Moreover, interactions between different metal centers can modulate the local electronic environment and facilitate the adsorption and transformation of oxygen-containing intermediates [10,11]. Among transition metal oxides, NiMoO_4_ have surfaced as standout OER catalysts. Their distinct electronic structures and synergistic effects enable high-performance water splitting by facilitating OER with exceptional efficiency, positioning them as viable candidates for sustainable energy systems [1,12]. The unique crystallographic arrangement and tailored electronic structure of NiMoO_4_ make it a premier candidate for advanced electrocatalysis. Recent studies emphasize that the multivalent nature of Mo centers creates a density of active sites that effectively lower the energy barrier for H_2_O adsorption and subsequent dissociation [13,14]. Concurrently, the integration of Ni not only modulates the local electronic environment to enhance charge-transfer kinetics but also reinforces the structural framework, leading to superior electrochemical stability even under rigorous operating conditions [15,16]. For example, NiMoO_4_/CP/APPJ-60 s [17], N-NiMoO_4_/NiS_2_ hybrids [18], P/Cr60-NiMoO_4_ [19], sulfur-doped NiMoO_4_/nickel foam (NF) [20], and Pt-MoO_3_/NiMoO_4_ [21]. Despite the potential of modifying NiMoO_4_ to overcome its intrinsic limitations, achieving high activity and long-term stability via a scalable, practical method remains a challenge. Addressing these gaps is essential for developing efficient catalysts suitable for industrial water-splitting applications.

Polymers are special molecules that can change surface properties and naturally organize themselves into structured arrangements in solution or at interfaces [22]. Among them, sodium dodecyl sulfate (SDS) is widely used because it easily forms stable micelles and interacts strongly with other species [23,24]. These micelles act like soft templates, helping to control how materials grow and develop. In addition, its negatively charged groups attract metal ions, allowing them to gather in specific regions and form more active sites, which ultimately improves the performance of the material. For example, an SDS-intercalated CoFe-layered double hydroxide (LDH)/FeOOH hierarchical electrocatalyst synthesized via a hydrothermal route demonstrated an overpotential of 290 mV at a current density of 10 mA cm^−2^ for the OER [25]. Similarly, recent studies on two-dimensional (2D) ultrathin Ni(OH)_2_ nanosheets modified with SDS have revealed that the formation of a Ni(OH)_2_-SDS heterojunction significantly alters the coordination environment of active sites, leading to enhanced catalytic activity with a reduced overpotential of 285 mV at 10 mA cm^−2^ [26]. Furthermore, the intercalation of SDS into trimetallic NiFe-La-LDH systems has also been reported to improve OER performance. The resulting NiFe-La-SDS-LDH electrocatalyst exhibits a notably low overpotential of 230 mV at a current density of 10 mA cm^−2^ [27].

Motivated by the aforementioned considerations, a series of NiMoO_4_-SDS electrocatalysts were synthesized via a facile hydrothermal approach to investigate the influence of SDS-assisted growth regulation on the structural, morphological, and electrochemical characteristics of NiMoO_4_. Among the investigated compositions, NiMoO_4_-SDS-2 demonstrated the most favorable OER performance. The present study highlights SDS-assisted growth regulation as a promising strategy for tailoring NiMoO_4_ nanostructures toward enhanced alkaline OER performance.

## 2. Experimental Section

### 2.1. Chemicals

Nickel nitrate hexahydrate (Ni(NO_3_)_2_·6H_2_O) and sodium dodecyl sulfate (SDS, C_12_H_25_NaSO_4_) were purchased from Sigma-Aldrich (St. Louis, MO, USA). Potassium hydroxide (KOH, >85%) was obtained from DaeJung Chemicals & Metals (Siheung-si, Gyeonggi-do, Republic of Korea). Ammonium heptamolybdate tetrahydrate ((NH_4_)_6_Mo_7_O_24_·4H_2_O, 99.0%) was supplied by Fisher Chemical (Gangnam-gu, Seoul, Republic of Korea). Nickel foam (NF) substrates used for electrode preparation were procured from NARA Cell-Tech Corporation (Geumcheon-gu, Seoul, Republic of Korea). All reagents were of analytical grade and used as received without further purification. Deionized (DI) water was employed throughout all experimental procedures to ensure reproducibility and reliability of the results.

### 2.2. Preparation of Pristine NiMoO_4_ and NiMoO_4_-SDS Materials

NiMoO_4_ nanostructures were synthesized via a controlled hydrothermal approach. In a typical procedure, 0.013 g of Ni(NO_3_)_2_·6H_2_O (~0.0447 mmol precursor) and 0.055 g of (NH_4_)_6_Mo_7_O_24_·4H_2_O (~0.0445 mmol precursor) were dissolved in 30 mL of deionized water under continuous magnetic stirring to form a homogeneous precursor solution. The resulting solution was then transferred into a 50 mL Teflon-lined stainless-steel autoclave and maintained at 150 °C for 8 h to facilitate the hydrothermal reaction. After naturally cooling to room temperature, the NF substrate with the deposited product was collected, thoroughly rinsed with deionized water and ethanol to remove residual impurities, and dried at 60 °C. The dried samples were subsequently annealed in air at 450 °C for 2 h to obtain well-crystallized NiMoO_4_.

**Figure 1:**
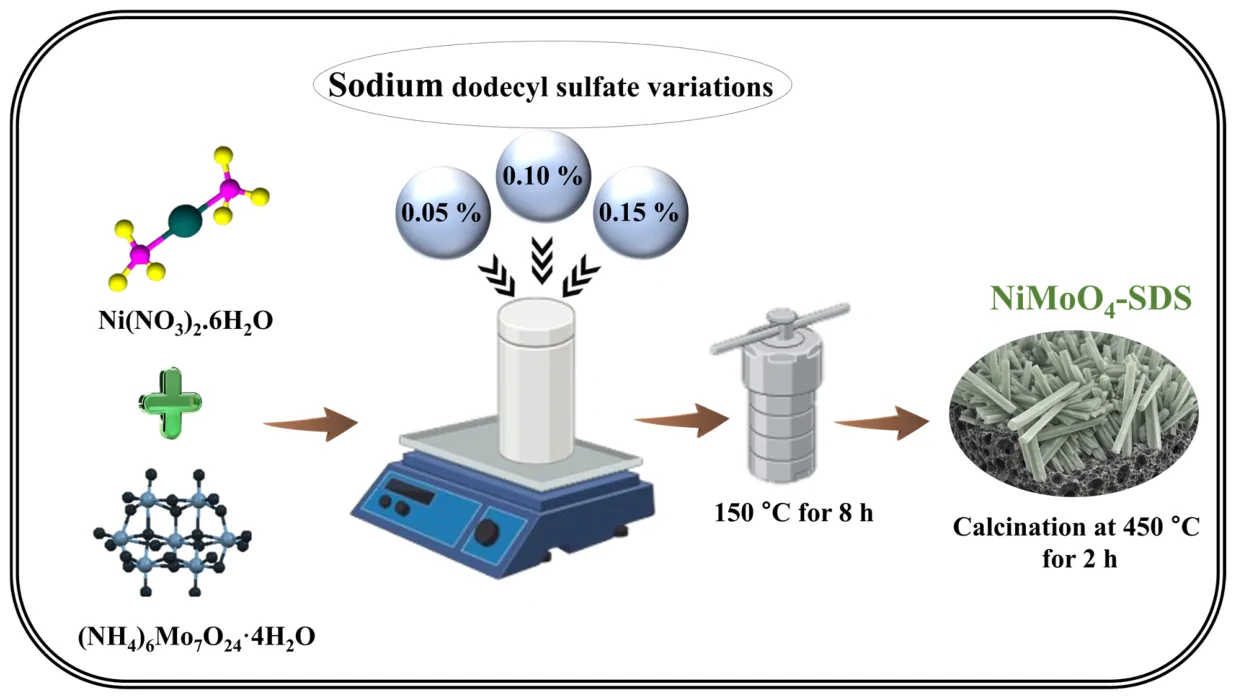
Schematic representation of synthesis of NiMoO_4_-SDS.

For the preparation of SDS-modified NiMoO_4_ composites, predetermined amounts of SDS were introduced into the precursor solution containing NiMoO_4_, followed by ultrasonication for 10 min to ensure uniform dispersion and effective interaction between the surfactant and precursor species. Three different SDS concentrations (0.05, 0.10, and 0.15%) were employed to synthesize a series of composites, which were denoted as NiMoO_4_-SDS-1, NiMoO_4_-SDS-2, and NiMoO_4_-SDS-3, respectively. A schematic illustration of the synthesis process is presented in Figure 1, depicting the stepwise formation in directing the morphological evolution of the nanostructures.

### 2.3. Physicochemical Characterization

The crystalline/amorphous nature of the as-prepared samples was studied using an X-ray diffraction (XRD; X’Pert Pro diffractometer equipped with Cu Kα radiation as the X-ray source) pattern (PANalytical, Almelo, The Netherlands). X-ray photoelectron spectroscopy (XPS; Thermo Scientific K-α spectrometer, Cheshire, UK analysis system) exposed the elemental composition and oxidation state of the elements. SEM (HITACHI S-4800, Tokyo, Japan) equipped with an energy dispersive x-ray analyzer (EDAX, Tokyo, Japan) was employed to explore the surface phenomenon of the as-prepared materials.

### 2.4. Electrochemical Characterization

Electrochemical measurements were carried out using a Biologic WBCS3000 battery cycler (Gieres, France) in a standard three-electrode configuration. Nickel foam served as the substrate for the working electrode. Prior to use, the NF was thoroughly cleaned by ultrasonication in deionized water, acetone, and ethanol for 20 min each, followed by drying at 60 °C overnight to ensure a clean and active surface. A platinum plate and Ag/AgCl electrode were employed as the counter and reference electrodes, respectively, while 1.0 M KOH was used as the electrolyte for all measurements. For an active electrode area of 1 × 1 cm^2^, the catalyst loadings of NiMoO_4_, NiMoO_4_-SDS-1, NiMoO_4_-SDS-2, and NiMoO_4_-SDS-3 on NF were determined to be approximately 0.38, 0.35, 0.31, and 0.32 mg cm^−2^, respectively. The OER performance was evaluated using linear sweep voltammetry (LSV) at a scan rate of 5 mV s^−1^. Cyclic voltammetry (CV) was conducted in the non-faradaic region at varying scan rates (5–20 mV s^−1^) to estimate the double-layer capacitance (C_dl_), which is directly related to the electrochemically active surface area (ECSA). The ECSA was calculated using the relation ECSA = C_dl_/C_s_, where C_s_ represents the specific capacitance of a flat surface in 1.0 M KOH (typically 0.040 mF cm^−2^).

The durability of the electrocatalysts was assessed through accelerated cycling tests involving 5000 CV cycles, with stability evaluated by comparing LSV curves recorded before and after cycling. Long-term operational stability was further examined using chronopotentiometry (CP) under constant current conditions. Additionally, electrochemical impedance spectroscopy (EIS) measurements were performed with an AC amplitude of 10 mV over a frequency range of 100 kHz to 0.1 Hz.

## 3. Result and Discussion

**Figure 2:**
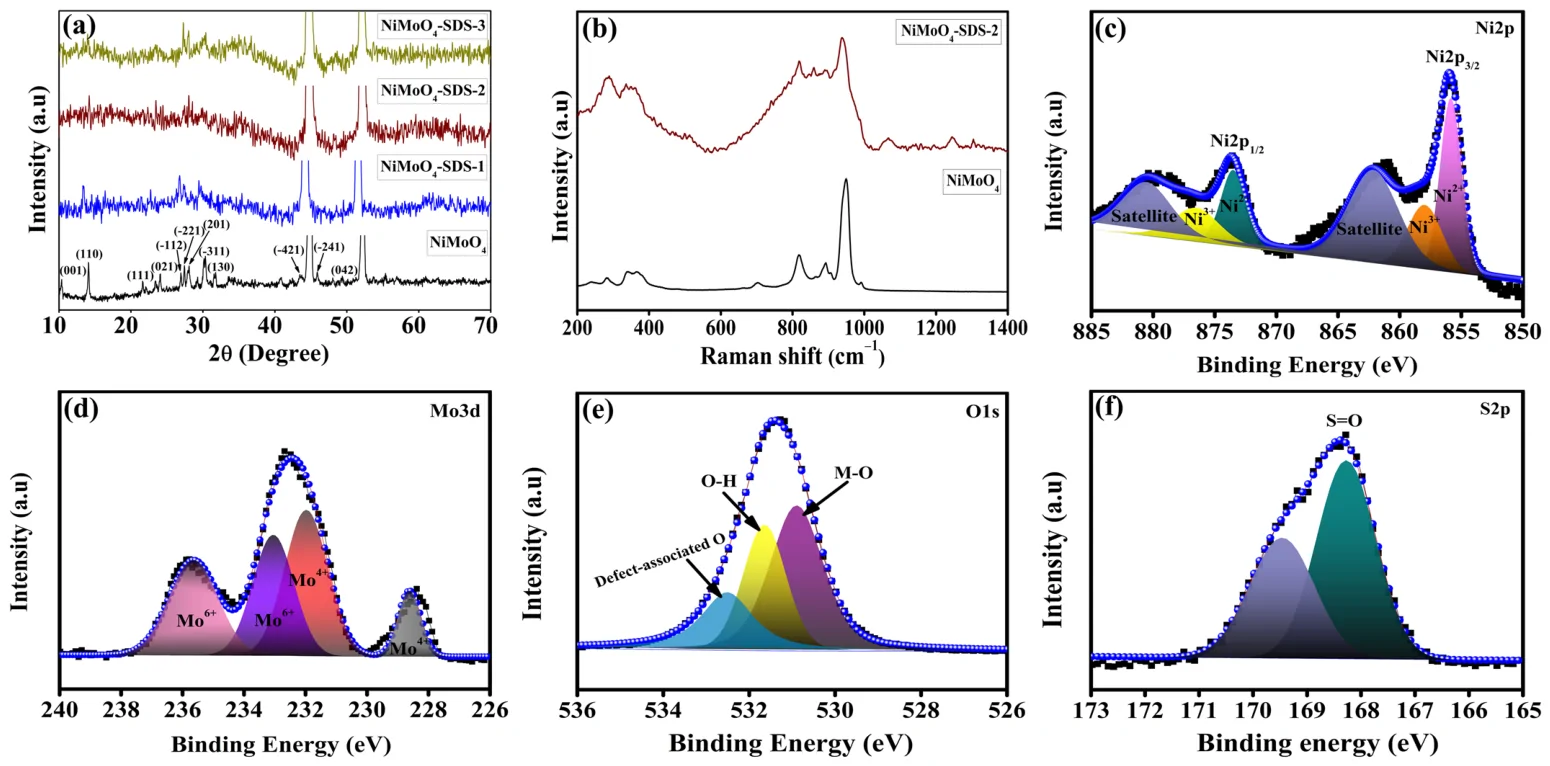
(**a**) XRD spectra of all the electrocatalysts. (**b**) Raman spectra of NiMoO_4_ and NiMoO_4_-SDS-2 electrocatalysts; and high-resolution XPS spectra of (**c**) Ni2p, (**d**) Mo3d, (**e**) O1s, and (**f**) S2p of the NiMoO_4_-SDS-2 electrocatalyst.

X-ray diffraction (XRD) analysis of pristine NiMoO_4_, NiMoO_4_-SDS-1, NiMoO_4_-SDS-2, and NiMoO_4_-SDS-3 composites were presented in Figure 2a. The diffraction pattern of pristine NiMoO_4_ exhibits well-defined and sharp peaks located at 10.3°, 14.0°, 21.6°, 24.1°, 26.9°, 27.4°, 28.0°, 30.3°, 31.7°, 43.4°, 45.8°, and 49.3°, corresponding to the (001), (110), (111), (021), (−112), (−221), (201), (−311), (130), (−421), (−241), and (042) planes, respectively. These reflections are in excellent agreement with the standard JCPDS card No. 01-086-0361, confirming the formation of highly crystalline monoclinic-phase NiMoO_4_ [28,29]. Following SDS-assisted synthesis, the characteristic diffraction features of NiMoO_4_ are well preserved, demonstrating the retention of the primary crystalline phase. Meanwhile, subtle variations in the diffraction peak positions and relative intensities are observed across the SDS-assisted samples, indicating that SDS influences the crystallization and growth characteristics of NiMoO_4_. The interplanar spacing (d-spacing) was calculated using Bragg’s equation:

nλ = 2dsinθ(1)

where λ represents the X-ray wavelength, θ is the diffraction angle, and n is the diffraction order [30,31]. The pristine NiMoO_4_ exhibits a d-spacing of 6.27 Å for the (110) plane, whereas the NiMoO_4_-SDS-2 composite shows a slightly reduced value of 6.23 Å for the same plane. This refined variation in interplanar spacing further reflects the influence of SDS-assisted growth on the local structural characteristics of NiMoO_4_.

Raman spectroscopy shown in Figure 2b was employed to examine the vibrational characteristics and local structural environment of pristine NiMoO_4_ and NiMoO_4_-SDS-2. The Raman spectrum of pristine NiMoO_4_ exhibits several characteristic vibrational modes associated with the molybdate framework. The band observed at approximately 281 cm^−1^ is assigned to the deformation vibration of the Mo-O-Mo linkage. The bands appearing within 339–366 cm^−1^ are attributed to the bending vibrations of Mo-O units. At higher wavenumbers, the characteristic band located near 702 cm^−1^ corresponds to the stretching vibration of the Ni-O-Mo linkage, reflecting the metal–oxygen connectivity within the NiMoO_4_ lattice. The band at approximately 817 cm^−1^ is associated with a Mo-O-related bending/vibrational mode. Furthermore, the prominent Raman bands centered at 893 and 949 cm^−1^ are assigned to the symmetric and asymmetric stretching vibrations of terminal Mo=O bonds, respectively. These characteristic vibrational features collectively support the successful formation of the NiMoO_4_ phase [18,32,33]. Following SDS-assisted modification, the NiMoO_4_-SDS-2 sample retains the major characteristic vibrational features of NiMoO_4_, suggesting that the fundamental metal-oxide framework remains preserved. Nevertheless, an overall reduction in the intensity and broadening of several NiMoO_4_-related Raman bands are observed, indicating increased local structural disorder and perturbation of the metal-oxygen coordination environment [34]. In addition to the NiMoO_4_-related vibrations, NiMoO_4_-SDS-2 displays additional Raman features at higher wavenumbers. In ascending Raman-shift order, the newly observed band at approximately 859 cm^−1^ is attributed to C-O-SO_3_-related stretching vibrations, while the feature near 1066 cm^−1^ corresponds to the symmetric stretching vibration of the SO_3_ headgroup. The band appearing at approximately 1245 cm^−1^ is associated with an S=O-related stretching vibration, whereas the feature at around 1304 cm^−1^ is assigned to the CH_2_ twisting mode of the hydrocarbon chain [35,36]. The emergence of these SDS-associated vibrational features, together with the preservation of the characteristic NiMoO_4_ modes, provides evidence for the presence of SDS-derived species associated with the NiMoO_4_-SDS-2 material.

The high-resolution X-ray photoelectron spectroscopy (XPS) was elucidated in Figure 2. The Ni2p spectrum (Figure 2c) exhibits two well-defined spin–orbit doublets. The prominent peaks centered at 855.8 eV and 857.9 eV are assigned to Ni^2+^ and Ni^3+^ species of the Ni2p_3/2_ level, respectively, while the corresponding peaks at 873.4 eV and 875.8 eV originate from Ni^2+^ and Ni^3+^ states of the Ni2p_1/2_ level, with the distinct satellite features at 862.9 eV and 880.4 eV, respectively [37,38]. The Mo3d high-resolution spectrum shown in Figure 2d reveals two sets of characteristic doublets, indicating multiple oxidation states of molybdenum. The peaks located at 228.9 eV and 231.9 eV correspond to the Mo^4+^ 3d_5/2_ and 3d_3/2_ states, respectively, whereas the higher binding energy peaks at 232.9 eV and 235.6 eV are attributed to the Mo^6+^ 3d_5/2_ and 3d_3/2_ states [38]. Furthermore, the O1s spectrum (Figure 2e) provides insight into the oxygen-related species present in the material. The dominant peak at 530.50 eV is associated with lattice oxygen in metal-oxygen (M-O) bonds, confirming the formation of the oxide framework. The additional peaks observed at 531.37 eV and 532.18 eV are attributed to defect-associated oxygen species and surface hydroxyl groups (O-H), respectively [39,40]. The characteristic S2p peaks observed at 168.2 and 169.4 eV (Figure 2f) are assigned to the S=O bonding environment of SDS-derived anionic species, providing evidence for the presence of SDS-related sulfur species within the composite structure [41,42].

**Figure 3:**
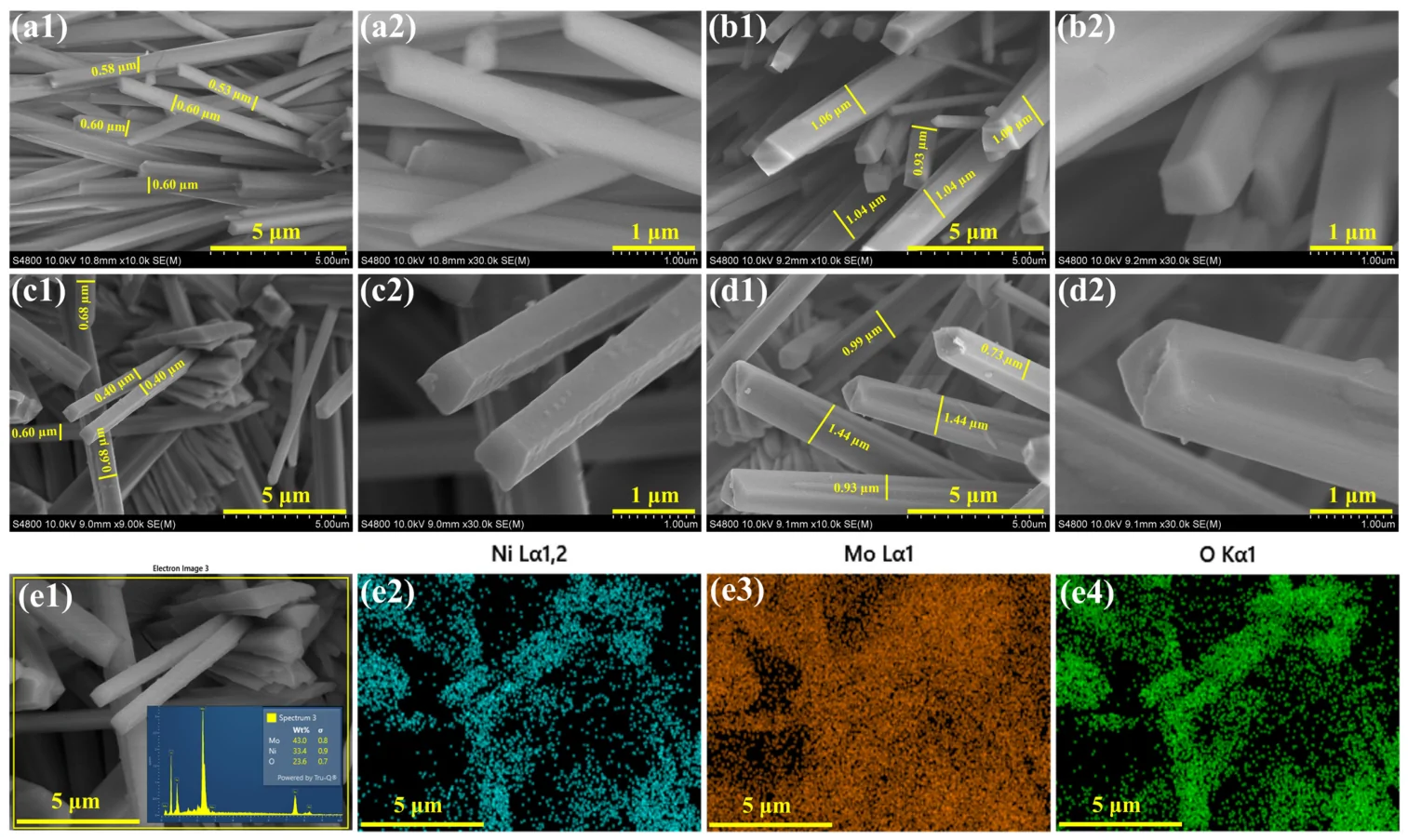
SEM images of (**a1**,**a2**) NiMoO_4_, (**b1**,**b2**) NiMoO_4_-SDS-1, (**c1**,**c2**) NiMoO_4_-SDS-2, and (**d1**,**d2**) NiMoO_4_-SDS-3 electrocatalysts. (**e1**–**e4**) Energy-dispersive X-ray spectroscopy analysis and elemental mapping data of the NiMoO_4_-SDS-2 electrocatalyst.

The surface morphology and structural evolution of pristine NiMoO_4_ and SDS-assisted samples were investigated by SEM, as presented in Figure 3. Pristine NiMoO_4_ (Figure 3(a1,a2)) exhibits a dense assembly of elongated nanorod-like structures with relatively smooth surfaces and random orientation. Following the introduction of SDS, noticeable changes in the morphology are observed. NiMoO_4_-SDS-1 (Figure 3(b1,b2)) retains the rod-like architecture, while the structures appear more distinctly developed with improved separation compared with the pristine sample, indicating the influence of SDS on the nucleation and growth behavior of NiMoO_4_. In NiMoO_4_-SDS-2 (Figure 3(c1,c2)), a more uniformly distributed nanorod network with increased inter-rod spacing is observed, resulting in a comparatively open architecture. Such morphological characteristics may facilitate electrolyte accessibility and increase the exposure of electrochemically accessible surface sites. NiMoO_4_-SDS-3 (Figure 3(d1,d2)) also retains its rod-like morphology, although relatively thicker and more closely arranged structures are observed, indicating a concentration-dependent influence of SDS on the growth process. Quantitative analysis of the SEM images revealed average feature sizes of approximately 0.58, 1.03, 0.55, and 1.10 µm for NiMoO_4_, NiMoO_4_-SDS-1, NiMoO_4_-SDS-2, and NiMoO_4_-SDS-3, respectively. Notably, NiMoO_4_-SDS-2 exhibits the smallest average size of 0.55 µm, indicating the effective role of the optimized SDS concentration in regulating the growth and dimensional characteristics of the NiMoO_4_ nanostructures.

The elemental composition and even distribution of the optimized NiMoO_4_-SDS-2 sample were analyzed using energy-dispersive X-ray spectroscopy (EDAX) and corresponding elemental mapping, as presented in Figure 3(e1–e4). The EDAX spectrum in Figure 3(e1) confirms the presence of Ni, Mo, and O as the primary constituent elements, with 33.4, 43.0, and 23.6 Wt%, indicating the high purity of the synthesized material. The elemental mapping images provide deeper insight into the distribution of these elements across the nanostructured surface. As shown in Figure 3(e2–e4), the individual maps corresponding to Ni, Mo, and O exhibit a uniform and homogeneous distribution throughout the entire structure. The consistent dispersion of all elements without noticeable segregation or clustering confirms the successful integration of the components at the nanoscale.

**Figure 4:**
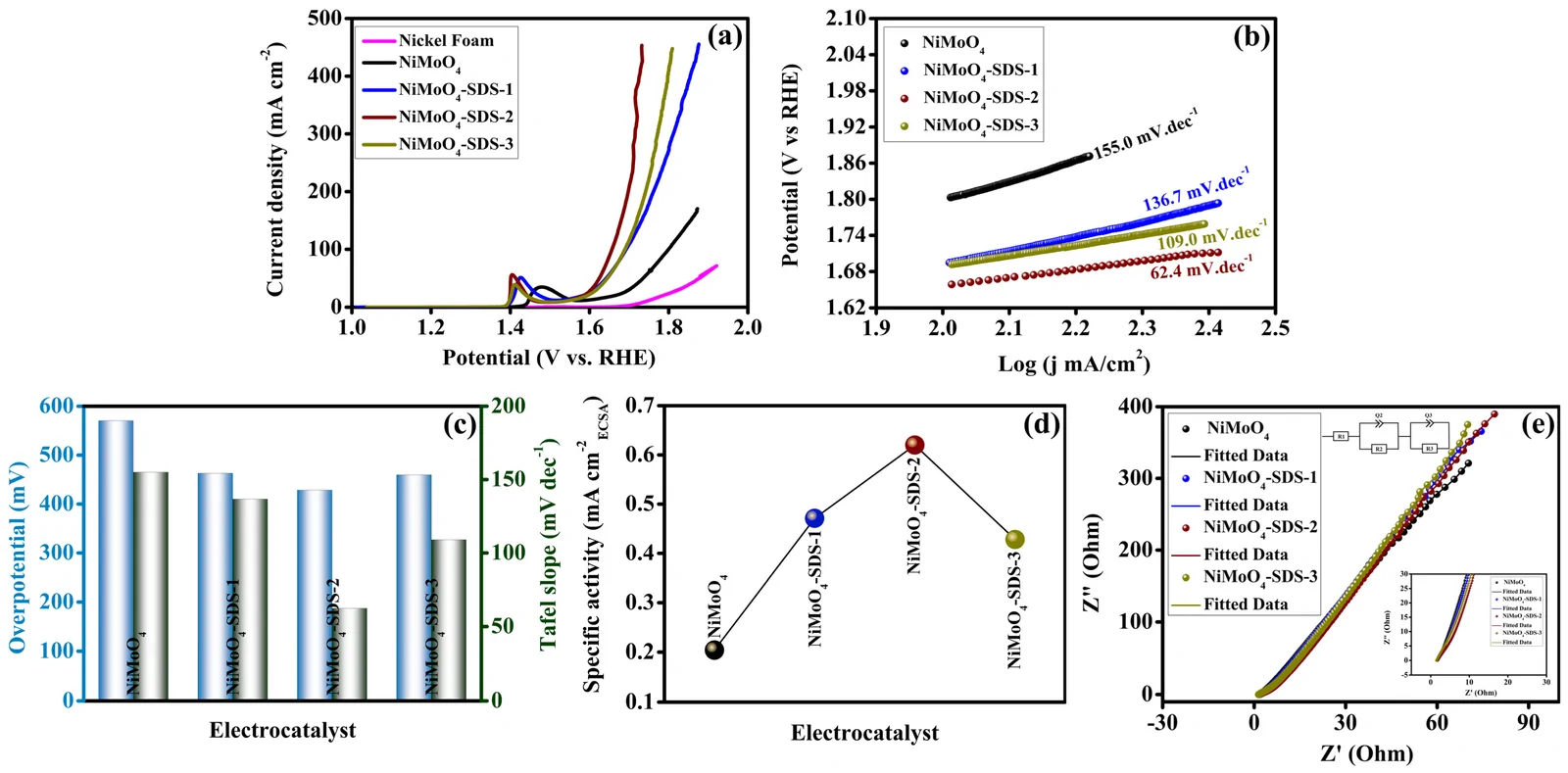
Electrochemical characterizations of electrocatalyst OER performances: (**a**) LSV curves at 5 mV s^−1^ scan rate, (**b**) Tafel slopes, (**c**) the OER performance relating to overpotential at 10 mA cm^−2^ and Tafel slope, (**d**) specific activity, and (**e**) EIS spectra of all the electrocatalysts.

The electrocatalytic oxygen evolution reaction performance of pristine NiMoO_4_ and SDS-modified samples (NiMoO_4_-SDS-1, NiMoO_4_-SDS-2, and NiMoO_4_-SDS-3) was thoroughly evaluated using a conventional three-electrode configuration in 1.0 M KOH electrolyte. To minimize the influence of uncompensated ohmic resistance, iR correction was applied to the polarization data of all electrocatalysts. The linear sweep voltammetry (LSV) curves presented in Figure 4a and Figure 4c reveal that the overpotentials required to achieve a current density of 100 mA cm^−2^ are 570.3, 462.2, 428.5, and 459.6 mV for NiMoO_4_, NiMoO_4_-SDS-1, NiMoO_4_-SDS-2, and NiMoO_4_-SDS-3, respectively. In contrast, bare nickel foam exhibited substantially lower OER activity and did not attain a current density of 100 mA cm^−2^ within the investigated potential window. Among these, the NiMoO_4_-SDS-2 electrode exhibits the lowest overpotential which is arises from its modified local chemical environment and enhanced active site exposure, which facilitate faster charge transfer and improved OER kinetics. To further elucidate the reaction kinetics, Tafel analysis was performed based on the LSV data. The Tafel slope serves as an important kinetic parameter, providing insight into the rate-determining step and intrinsic catalytic efficiency of the electrode material. As illustrated in Figure 4b,c, the Tafel slopes were determined to be 155.0, 136.7, 62.4, and 109.0 mV dec^−1^ for NiMoO_4_, NiMoO_4_-SDS-1, NiMoO_4_-SDS-2, and NiMoO_4_-SDS-3, respectively. The lowest Tafel slope observed for NiMoO_4_-SDS-2 confirms its faster reaction kinetics and superior electrocatalytic behavior. The improved performance can be attributed to modifications in microstructure and surface chemistry induced by SDS incorporation, which facilitate enhanced exposure of active sites and improved electron transport pathways. To evaluate the intrinsic electrocatalytic activity while minimizing the influence of differences in accessible surface area, the OER current density was normalized with respect to the electrochemically active surface area (ECSA). The corresponding specific activities (Figure 4d) were calculated as 20.4 × 10^−2^, 47.1 × 10^−2^, 62.0 × 10^−2^, and 42.8 × 10^−2^ mA cm^−2^_ECSA_ for NiMoO_4_, NiMoO_4_-SDS-1, NiMoO_4_-SDS-2, and NiMoO_4_-SDS-3, respectively. As shown in Figure, NiMoO_4_-SDS-2 exhibits the highest ECSA-normalized activity, demonstrating that its enhanced OER performance extends beyond differences in electrochemically accessible surface area and reflects improved activity of the accessible catalytic surface. Electrochemical impedance spectroscopy (EIS) measurements (Figure 4e) further support this observation. In the Nyquist plots, R_s_ represents the solution resistance, Q_2_ and Q_3_ represent constant phase elements (CPEs), while R_ct_ corresponds to the charge transfer resistance at the electrode-electrolyte interface. Notably, the NiMoO_4_-SDS-2 electrode exhibits the lowest R_ct_ value (5.8 Ω) compared to the NiMoO_4_ (11.14 Ω), NiMoO_4_-SDS-1 (8.59 Ω), and NiMoO_4_-SDS-3 (7.49 Ω), indicating reduced interfacial resistance and more efficient charge transfer during the OER process.

**Figure 5:**
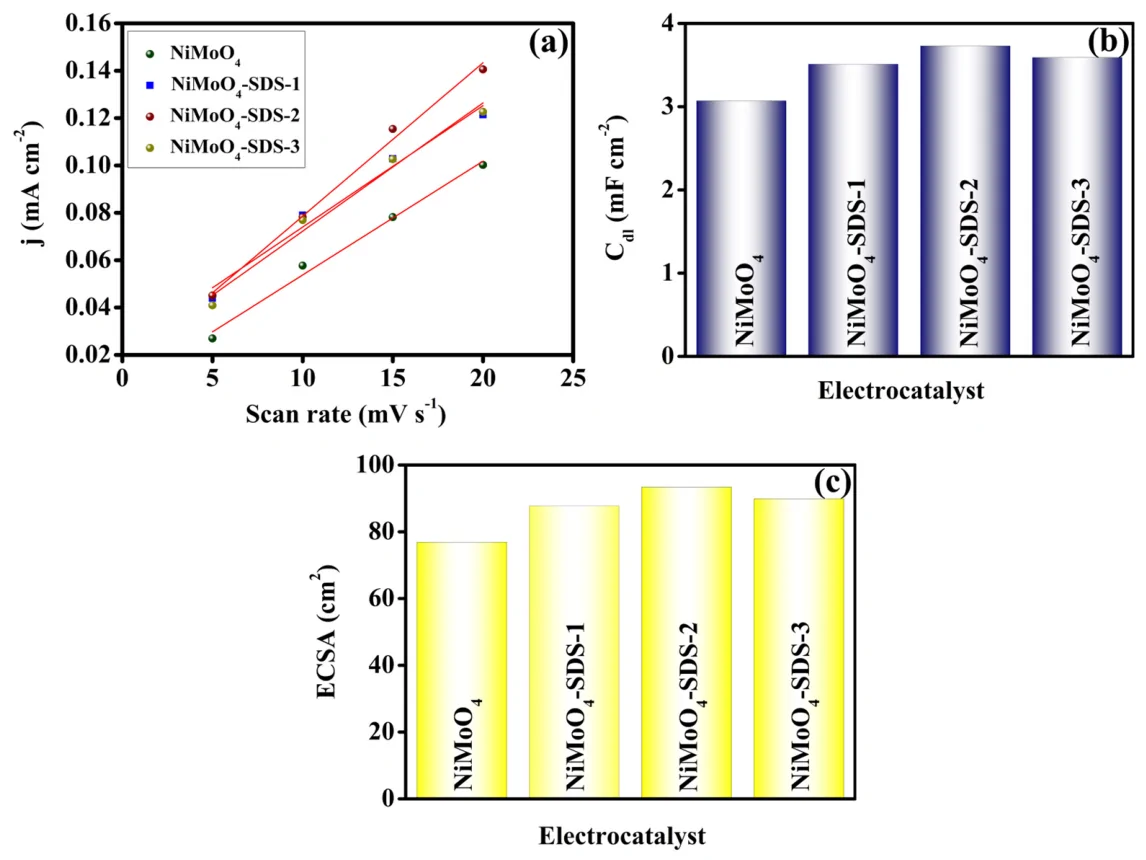
(**a**) 2C_dl_ graph, (**b**) C_dl_ graph, and (**c**) ECSA graph of all the electrocatalysts at different scan rates.

The electrochemically active surface area (ECSA) of the catalysts was estimated through the evaluation of double-layer capacitance (C_dl_), which serves as a reliable indicator of the number of accessible active sites for the OER. Since C_dl_ is directly proportional to ECSA, higher capacitance values reflect a greater density of electrochemically active surface sites. Cyclic voltammetry (CV) measurements were conducted within the non-faradaic potential region at various scan rates to determine C_dl_. The capacitive current density difference (Δj = j_a_ − j_c_), obtained from the anodic (j_a_) and cathodic (j_c_) currents, was plotted as a function of scan rate, and the slope of the resulting linear fit corresponds to 2C_dl_ (Figure 5a), from which C_dl_ is derived. As shown in Figure 5b, the NiMoO_4_-SDS-2 electrode exhibits the highest C_dl_ value of 3.73 mF cm^−2^, compared to 3.07, 3.51, and 3.59 mF cm^−2^ for pristine NiMoO_4_, NiMoO_4_-SDS-1, and NiMoO_4_-SDS-3, respectively. This indicates that NiMoO_4_-SDS-2 possesses a larger electrochemically active surface area and a higher density of accessible catalytic sites. The corresponding ECSA values, presented in Figure 5c, further confirm this trend, with NiMoO_4_, NiMoO_4_-SDS-1, NiMoO_4_-SDS-2, and NiMoO_4_-SDS-3 exhibiting ECSA values of 76.87, 87.75, 93.37, and 89.87 cm^2^, respectively. These results clearly demonstrate that the NiMoO_4_-SDS-2 catalyst provides the most favorable surface characteristics for enhanced electrocatalytic performance.

**Figure 6:**
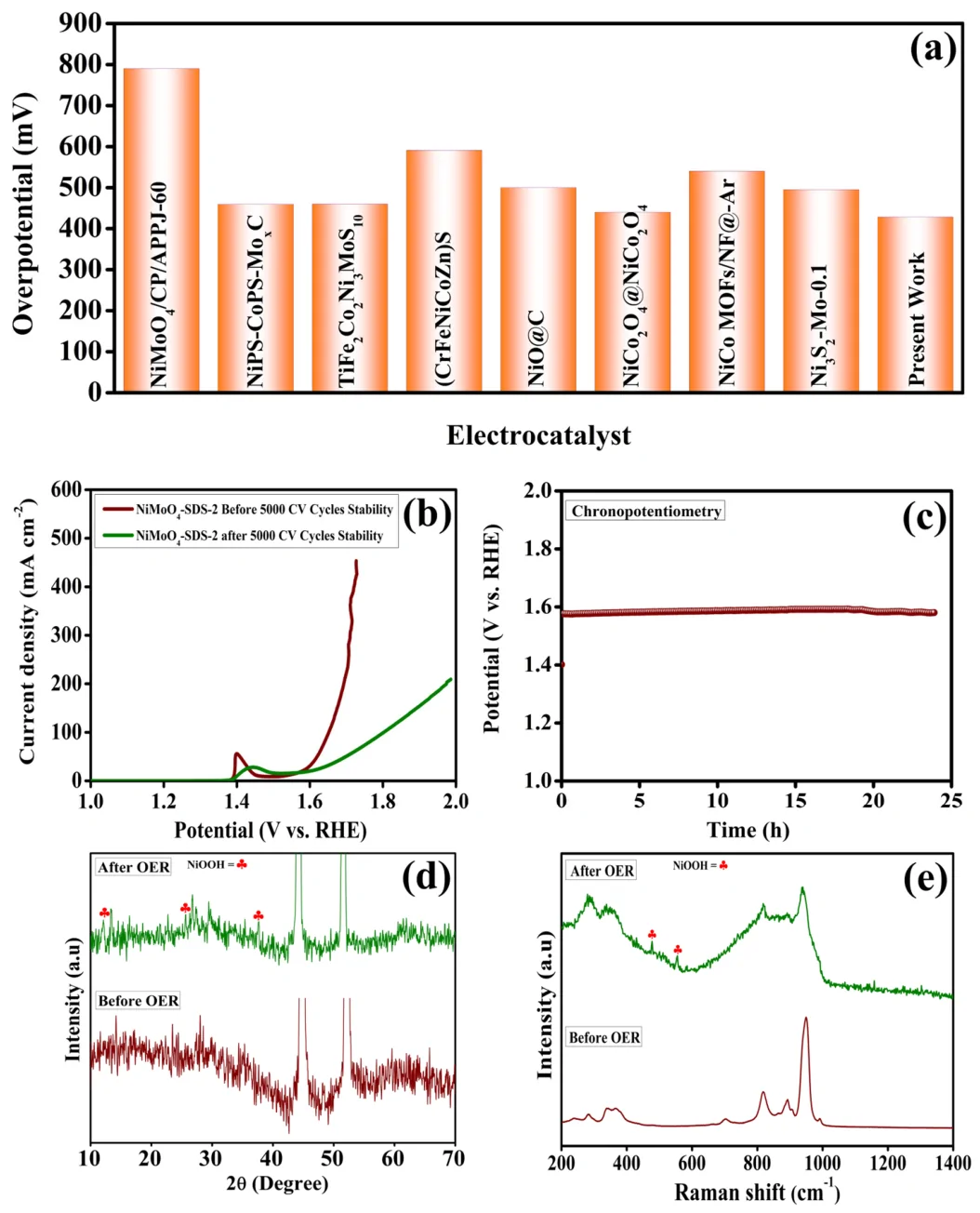
(**a**) Comparative OER overpotential results of NiMoO_4_-SDS-2 with other reported electrocatalysts, (**b**) LSV curves of NiMoO_4_-SDS-2 before and after 5000 CV cycles, and (**c**) chronopotentiometry study. Post-OER analysis of (**d**) XRD spectra and (**e**) Raman spectra of NiMoO_4_-SDS-2.

The comparative analysis of NiMoO_4_-SDS-2 with recently reported electrocatalysts in Figure 6a and Table 1 clearly demonstrates its superior OER performance. The optimized catalyst exhibits enhanced activity, reflected by its lower overpotential and improved reaction kinetics relative to previously reported systems. Moreover, SDS-assisted structural regulation may contribute to favorable interfacial charge-transfer characteristics and facilitate the electrochemical processes associated with OER, thereby enhancing the overall electrocatalytic performance. The long-term stability of an electrocatalyst is a critical parameter for evaluating its practical applicability in electrochemical energy systems. To investigate the durability of the NiMoO_4_-SDS-2 electrode, both cyclic voltammetry (CV) cycling and chronopotentiometric measurements were carried out in 1.0 M KOH electrolyte. The CV stability test was performed at a scan rate of 50 mV s^−1^ to assess the structural and electrochemical robustness under repeated redox cycling. As shown in Figure 6b, the overpotential required to achieve 100 mA cm^−2^ increased from 428.5 mV before cycling to 572.9 mV after 5000 CV cycles, corresponding to an overpotential increase of 144.4 mV. This variation indicates a measurable decline in OER activity after prolonged cycling. In addition, long-term operational durability was evaluated using chronopotentiometry at a constant current density of 10 mA cm^−2^ over a continuous period of 24 h (Figure 6c). The NiMoO_4_-SDS-2 electrode demonstrates a highly stable potential profile with no significant fluctuation throughout the test duration, confirming its strong resistance to performance decay under sustained OER conditions. To investigate the structural evolution of NiMoO_4_-SDS-2 under OER conditions, post-OER XRD and Raman analyses were performed. As shown in Figure 6d, the principal diffraction features of the initial NiMoO_4_ phase remain detectable after OER testing, suggesting that a portion of the underlying crystalline framework is retained. However, the pronounced attenuation of several characteristic NiMoO_4_ reflections indicates substantial electrochemically driven structural reconstruction during OER. Notably, new diffraction features appear at approximately 12.1°, 25.6°, and 37.6°, which can be indexed to the (003), (006), and (102) planes of NiOOH, respectively, in accordance with JCPDS No. 00-006-0075 [43]. Further evidence for this reconstruction is provided by the post-OER Raman spectrum presented in Figure 6e. Two prominent vibrational bands centered at approximately 477 and 555 cm^−1^ are observed and assigned to the characteristic E_g_ and A_1g_ vibrational modes of NiOOH species, respectively [44,45]. Post-OER XRD and Raman analyses confirm NiOOH formation while indicating partial retention of the NiMoO_4_ crystalline framework.

## 4. Conclusions

In this study, a series of NiMoO_4_-based electrocatalysts were successfully synthesized through a facile hydrothermal approach using different concentrations of SDS as a growth-regulating agent. Structural and morphological analyses revealed the formation of well-defined nanorod architectures, with SDS-assisted synthesis effectively regulating the morphology and surface characteristics of NiMoO_4_. Among the investigated electrodes, NiMoO_4_-SDS-2 exhibited the best OER performance, requiring an overpotential of 428.5 mV to achieve 100 mA cm^−2^, compared with 570.3, 462.8, and 459.6 mV for pristine NiMoO_4_, NiMoO_4_-SDS-1, and NiMoO_4_-SDS-3, respectively. The enhanced activity of NiMoO_4_-SDS-2 was further supported by its favorable Tafel slope, lower charge-transfer resistance, higher ECSA, and superior ECSA-normalized specific activity, demonstrating that the OER enhancement is not solely governed by increased surface accessibility. Post-OER XRD and Raman analyses revealed the formation of NiOOH species while indicating partial retention of the parent NiMoO_4_ crystalline framework. Overall, the results demonstrate that SDS-assisted growth regulation provides an effective strategy for tailoring the morphological and electrochemical characteristics of NiMoO_4_ toward enhanced alkaline OER performance.

## Figures and Tables

**Table 1:** Comparison of existing electrocatalyst OER performance result with other reported electrocatalysts.

Electrocatalyst	Mass Loading (mg cm^−2^)	Electrolyte	Substrate	Overpotential (mV @ 100 mA cm^−2^)	Tafel (mV dec^−1^)	Ref.
NiMoO_4_/CP/APPJ-60 s	-	1 M KOH	CP	790	109.1	[17]
NiPS–CoPS–Mo_x_C	0.255	1 M KOH	GCE	459.2	66.2	[46]
TiFe_2_Co_2_Ni_3_MoS_10_	0.20	1 M KOH	GCE	460	53.9	[47]
(CrFeNiCoZn)S	1	1 M KOH	GCE	591	63	[48]
NiO@C	-	1 M KOH	NF	500	92	[49]
NiCo_2_O_4_@NiCo_2_O_4_	0.3	1 M KOH	NF	440	163	[50]
NiCo MOFs/NF@-Ar	4.02	1 M KOH	NF	540	480	[51]
Ni_3_S_2_-Mo-0.1	-	1 M KOH	NF	495	84.83	[52]
NiMoO_4_-SDS-2	0.31	1 M KOH	NF	428.5	62.4	Present work

## Data Availability

The data presented in this study are available on request from the corresponding author.
